# Metacognitive Therapy for Work-Related Stress: A Feasibility Study

**DOI:** 10.3389/fpsyt.2021.668245

**Published:** 2021-05-31

**Authors:** Stefano De Dominicis, Maiken Lykke Troen, Pia Callesen

**Affiliations:** ^1^Department of Nutrition, Exercise and Sports, University of Copenhagen, Copenhagen, Denmark; ^2^PTS—Psicoterapia Training School, Jesi, Italy; ^3^Metakognitiv Psykologklinik, Copenhagen, Denmark; ^4^Cektos—Center for Metakognitiv Terapi, Copenhagen, Denmark

**Keywords:** metacognitive therapy, work-related stress, blood pressure, anxiety, depression, COVID−19

## Abstract

About 25% of EU workers experience work-related stress for all or most of their working time, showing that work-related stress is a major cause of health problems for the EU population. This situation has been worsened even more by the COVID-19 restrictions embraced by employers worldwide. However, a timely and sustainable intervention protocol for treating such issues has not been developed yet. Thus, the present research shows a first effective attempt based on Metacognitive therapy (MCT) to solve this issue. MCT was practiced on four individuals suffering from chronic work-related stress. Primary outcome variables were general mental health, perceived stress, and blood pressure. Participants were assessed at multiple baselines before the start of therapy and then attended a 3- and 6-months follow-up after treatment termination. Results showed significant improvements in general mental health, perceived stress, and blood pressure in each client. Secondary outcome variables improved too—maladaptive coping strategies, avoidance behaviors, and depression symptoms—corroborating the main findings. At 3- and 6-month follow-up, results were maintained. The findings suggest that MCT might be a promising and sustainable intervention for work-related stress, although a metacognitive model for stress and large-scale RCTs need to be developed and carried out to further explore the effect of MCT on stress. Our results represent one of the first attempts to treat work-related stress via Metacognitive Therapy and support the feasibility of the treatment, both in terms of its efficacy and sustainability, in a historical moment in which work-related stress is increased worldwide because of the COVID-19 pandemic. Within such a realm, our feasibility study should be followed by larger and controlled studies that, if successful, would provide various stakeholders—including organizational and institutional decision-makers—with a solid, timely and cost-effective method to help the workforce coping with work-related stress.

## Introduction

Work-related stress (1) has been defined as a person's state accompanied by physical, psychological and/or social complaints or dysfunctions resulting from the feeling of being unable to bridge the perceived or objective gap with the work requirements/expectations placed on herself. As such, it is a major cause of health problems in the EU, where about 25% of workers say they experience work-related stress for all or most of their working time, which in turn affects their health negatively (2). One way to conceptualize work-related stress relates to the inherent characteristics of the job (3), but personal factors too [e.g., peronality traits; (4)] affects how work-related stressors impact a given person.

Although stress is not included in the official diagnostic manuals ICD-10 and DSM-5, there is an increasing public demand for evidence-based interventions specifically aimed at mitigating various forms of stress, such as work-related stress (5, 6), or even environmental hazard-related stress [see for example (7–10)]. Furthermore, the COVID-19 pandemic not only has significantly augmented the psychological health risks in the general population (11–13), it has also specifically increased the number and weight of possible stressors to the work landscape (14, 15). Therefore, a timely, cost-efficient and effective treatment of work-related stress is highly needed.

To date, psychological treatment of stress in the general population as well as in the workplace, is traditionally approached with: (a) interventions entailing Cognitive-Behavior Therapy (CBT) strategies and tools (16, 17)—such as, among others, relaxation techniques, cognitive restructuring techniques and social training—that would generally aim at changing stress mindset (18); (b) managerial and organizational interventions (19); and (c) group prevention seminars [(20); for a review see (21)]. However, especially in the workplace, a general lack of control groups or follow-ups limits the interpretation of the conclusions (22, 23). This mixed pattern of results and the urgent need for effective solutions call for further development of the psychological understanding and treatment of general and work-related (chronic) stress, at least from an applied clinical perspective. Following this line of research, a first tentative of studying the possible effects of work-focused therapy using a metacognitive intervention has been carried out showing promising results (24). Therefore, we tested the hypothesis of whether it would be possible to treat work-related stress specifically, with a Metacognitive Therapy-based intervention.

## Metacognitive Therapy as a Transdiagnostic Model of Treatment

Hence, treatment of work-related stress could be developed following a clear conceptualization of stress and an individualized approach. Therefore, its effective treatment could perhaps be achieved from an individual-centered transdiagnostic model of treatment. This approach can indeed offer a series of advantages: it would be cost-effective for the training of practitioners (25); it would provide patients with an intervention based on concurrent treatment of multiple disorders; it would account for various forms of psychological distress instead of being syndrome-specific (26); it would be applicable across a variety of organizational settings.

Metacognitive Therapy (henceforth, MCT), a transdiagnostic model that can account for various psychological symptoms and pathologies (27), could be a suitable framework to further understand and treat chronic stress. MCT is based on the Self-Regulatory Executive Function model [S-REF; (28)], focusing mainly on processes that result in pathological and persistent thinking. This is indeed the main characteristic of emotional suffering (29): according to the model, all psychological disorders are associated with a Cognitive Attentional Syndrome (CAS) consisting of mental processes (like worry, rumination, threat monitoring, and other dysfunctional coping strategies) that backfire and maintain psychological symptoms and all forms of emotional dysfunctions and disorders (28). The CAS is caused by negative metacognitive beliefs, such as uncontrollability (e.g., “my worry is uncontrollable”) and danger beliefs (e.g., “my worry can physically harm my body”), and positive metacognitive beliefs (e.g., “worry helps me to cope”).

This model postulates that stressed individuals, such as those suffering from work-related stress, would engage in the CAS and worry about different issues (e.g., stressful events occurred at work, a negative relationship with colleagues or one's own boss, performance delivery, etc.) which can further results in the maladaptive cognitions, eventually translating in the reinforcement of the CAS. This dysfunctional conceptual processing, such as ruminating about coping with colleagues, worrying about the next meeting, or arguing against the boss, could become the routine rather than the exception and then persist over time (28). Within MCT's lenses, stress is the result of CAS, which is maintained by metacognitive beliefs (30, 31).

## Current Study

To date, a considerable amount of evidence supports the link between metacognitive beliefs and a series of psychological disorders, such as anxiety (27, 32, 33), depression (27, 34–36), psychosis (30, 37), and PTSD (31). However, very few studies have explored this hypothesis applied to stress (24, 38), and further research should be carried out to develop a metacognitive model for stress and to test the effects of MCT on stress-related disorders. In this feasibility study, we provide a first brick of evidence in this realm by showing a first effective attempt based on Metacognitive Therapy to reduce work-related stress.

Based on a series of case studies using a multiple-baseline research design (26), our main contribution is not theory testing, but rather is theory-building heuristics (39), to be further tested in future research through, for example, experimental designs. The present contribution will: (a) allow for a rough description of a phenomenon still requiring future in-depth analysis; and (b) provide descriptions of the effects of a specific applied procedure, which appears very promising for the timely, effective and sustainable treatment of work-related stress.

### Aim and Hypotheses

The general aim of this study is to evaluate the effect of MCT in the treatment of Work-Related Stress (henceforth, MCT-WRS), and therefore in fostering return to work of treated clients. To reach this general aim, five operational hypotheses have been developed:

- H1: MCT-WRS will improve clients' general mental health from baseline (pre-treatment) to end of treatment (H1a), which in turn will remain stable from end of treatment to follow-up (H1b).
- H2: MCT-WRS will reduce clients' perceived stress from baseline (pre-treatment) to end of treatment (H2a), which in turn will remain stable from end of treatment to follow-up (H2b).
- H3: MCT-WRS will lower systolic blood pressure from baseline (pre-treatment) to end of treatment (H3a), which in turn will remain stable from end of treatment to follow-up (H3b).
- H4: MCT-WRS will lower diastolic blood pressure from baseline (pre-treatment) to end of treatment (H4a), which in turn will remain stable from end of treatment to follow-up (H4b).
- H5: a significant clinical change is expected in general mental health and perceived stress in each client.

Furthermore, to address the operational validity and feasibility of the treatment: (a) return to work is also considered as one of the main operational outcomes of this study—although no hypothesis was originally developed with reference to this outcome; and (b) we also report a qualitative analysis aiming at testing the feasibility of the treatment by reporting meaningful statements of the clients related to the feasibility and acceptability of MCT-WRS to treat their stress.

## Methods

### Design

Following prior research (35), the present study tests the effect of MCT for work related stress via a multiple baseline design across four participants. Each client went through a baseline period, which varied from 4 to 6 weeks, in which the client's symptoms were measured each week using standardized measures. Treatment begun if the baseline was stable, which was defined by results not having dropped more than two data points before start (see the “Procedure” section for a detailed description of the Assessment and Treatment procedures). This definition of baseline stability is based on previous research using the same multiple baseline design (35). In addition, the present study includes a 3- and 6-month follow-up to measure the stability of the effects of therapy over time. Four participants received MCT once a week for eight-to-ten sessions, every session lasting between 45 and 60 min. As no metacognitive model or treatment manual for stress exists, the protocol for Generalized Anxiety Disorder (GAD) was implemented and used as the treatment manual (27).

### Participants

Recruitment happened via social media and posters hung in general practitioner's clinics around Copenhagen, Denmark. Volunteers were asked if they wanted to participate in a research project regarding “Metacognitive Therapy for work related stress.” Before baseline measurements started, volunteers were screened to meet the following inclusion criteria:

- Work life to be very stressful [measured by Cohen's Perceived Stress Scale PSS-10; (40)];
- Having been on sick leave from work due to work related stress with no subsequent symptom recovery;
- Being 18–65 years old;
- Agreeing to remain stable on any current medication;

Exclusion criteria were:

- Suffering from depression, measured with Beck Depression Inventory-II (BDI-II) (41);
- Primary diagnosis of anxiety, depression, Obsessive-Compulsive Disorder (OCD), Post-Traumatic Stress Disorder (PTSD) or any other axis-1 diagnosis, as screened by Structured Clinical Interview for DSM-IV (SCID-I) (42, 43);
- Past history of other psychological treatment in the past 2 years, psychosis, suicidal, drug abuse;

The first four volunteers that met the above criteria were included in the study.

#### Client SH

SH was a 34 years old woman living with her boyfriend with no children. She had been sick suffering from stress and off work for 3 months when the treatment began and had felt stressed for the previous year. SH had taken antidepressant (30 mg Citaopram®) for 3 years, but took the same dose throughout the study, follow-up period included. SH had had 12 sessions of psychodynamic therapy several years ago. SH spend about 8 h a day worrying about other's expectations at work and her fear of being fired. SH spend a great deal of her time sleeping in order to escape her thoughts and worries and she rarely participated in social situations.

#### Client RL

RL is a 26 years old man living with his girlfriend. They did not have children. RL had been stressed for 3 years, but his stress level had heightened in the months prior to therapy. RL had not been to therapy before. He worried about 4 h a day, mainly about his performance and deadlines at work. In addition, he worried about his stress symptoms fearing they could lead to a heart attack or blood clot.

#### Client AJ

AJ is a 56 years old, single lady. She had been off work sick full-time for 9 months but by the time the therapy started she had returned to work although she still experienced severe stress symptoms. She spent about 5 h a day worrying about the amount of work she had to solve and also about whether she was “good enough” both at her job and in her private life. Furthermore, she worried that she would get a heart attack or a blood clot because of the worry and stress. AJ had been in cognitive behavioral therapy 4 years ago for 20 sessions and took antidepressant (60 mg Cymbalta®), which she continued taking during the treatment.

#### Client TM

TM is a 33 years old man. Up until 3 years before the study, he was running his own company, but he had shut it down and moved in with a family member because of stress. He had felt stressed the past 7 years before this study. TM worried for about 8 h a day about never getting well-again and how he should take care of himself in order to reduce the risk of relapse. Years back, TM had tried cognitive therapy for 3 years with four different therapists to reduce stress, but the therapies provided only small temporary positive effects.

### Procedure

#### Assessment

Potential participants in the study contacted the therapist ML directly via e-mail or telephone and were invited to an assessment interview. All volunteers were screened with SCID-I, BDI-II and interviewed by the therapist ML to be selected for the study based on the inclusion criteria. Individuals who met all criteria completed the Perceived Stress Scale (PSS) before baseline start. Blood Pressure (BP) was measured before the therapy, at the end of the therapy and at follow-up. Generalized Anxiety Disorder Scale-Revised (GADS-R), General Health Questionnaire 30 (GHQ-30), and BDI-II were completed on a weekly basis during the baseline period and additionally before every therapy session during treatment. At the last session and at 3- and 6-months follow-up, participants completed the full set of measurements—GADS-R, GHQ-30, BDI-II, BP, and PSS.

#### Treatment

The GAD treatment protocol of MCT (27) was adapted to stress, and specifically applied to work-related stress, on the basis of a 2-fold reason: (1) MCT is transdiagnostic and any disorder, including stress-related ones, should be treatable with the model; (2) work-related stress is likely to be expressed both as physical symptoms (such as raised blood pressure) and psychological symptoms such as anxiety, panic attacks, sleep disturbances, dysphoria, and restlessness (44–46), which resemble anxiety symptoms. Our MCT protocol for work related stress—according to the original GAD protocol—consisted of 8-to-10 sessions lasting 45–60 min, conducted by ML who is MCT-trained therapist (see “Training of the Therapist” section for details). Yet, the fundamental difference of our stress protocol from the GAD protocol was in the socialization phase [for details see (27)] were the metacognitive model explained how stress (rather than anxiety) is caused and maintained by worry and other maladaptive coping strategies like monitoring and avoidance, and that in order to overcome stress, the client would need to reduce time spend on these maladaptive coping strategies.

Broadly, the purpose of MCT treatment is to reduce the CAS and change the psychological mechanisms that develops and maintains it (27). In order to do so, the client has to learn to identify worry and rumination and the thoughts that trigger these dysfunctional cognitive patterns (27). Accordingly, the focus in therapy is not the thought content but the clients' metacognition. In fact, the therapist challenges clients' positive and negative metacognitive beliefs via Socratic dialogue and behavioral experiments. The therapy provides the client with a new set of responses, alternative to the CAS, to negative thoughts and emotions. One of the objects of the therapy is to eliminate undesirable coping strategies (e.g., thought suppression, which only will make the undesired thought more frequent in the long run). Another objective of MCT is to minimize, in the client, avoidance of both situations and thoughts. Thus, the therapy effectiveness of depends on whether CAS has been reduces so that meta-worry and meta-beliefs have changed permanently (27).

Specifically, the MCT protocol for work related stress consisted of 8-to-10 sessions lasting 45–60 min, conducted by a MCT-trained therapist. The therapy was terminated: (a) when the protocol had been completed thoroughly; (b) the time participants spend worrying had been reduced to a minimum; and (c) positive and negative metacognitive beliefs were under 10%. Therapy was not ended before the participant scored under five on the GHQ-30, which is the cut off for poor mental health (47). Moreover, the client had to agree upon terminating therapy before doing so.

#### Training of the Therapist

The therapist ML had been trained in MCT throughout half a year's internship, which included participation in metacognitive group therapy, metacognitive courses, and metacognitive education. In addition, the therapist had several years of experience with clients. The therapist followed metacognitive supervision and metacognitive seminars in Manchester run by Adrian Wells to develop her metacognitive skills. PC, who is a level 2 MCT-I therapist, educated and supervised the ML during the “metacognitive therapy for work related stress” (MCT-WRS) intervention.

### Measures

The following self-reported measures were collected, as well as an objective test of blood pressure. Participants were tested from the beginning of baseline and every week during treatment and additionally at 3 and 6 months follow up.

#### General Health Questionnaire 30

GHQ-30 is a 30-item, self-administered questionnaire designed to identify and measure mental health status (48), measured by a four-point likert-scale. The results from the GHQ-30 were calculated using a binary scoring system, which means that every item was computed on the basis of whether it was present or absent (0-0-1-1). The total score could range from zero to 30. Any score over five is viewed as high and indicates a clinically significant disorder. The questionnaire has shown high internal consistency, good reliability and good validity (47, 49). Furthermore, making this questionnaire particularly relevant for the present contribution, Jenkins (50) found that GHQ-30 has satisfying validity in work related contexts and that psychiatric disorders, as measured by the questionnaire, are significantly correlated with proxies of work-related stress.

#### Perceived Stress Scale

PSS is a standardized, 10-item, self-report questionnaire, which measures the degree to which participants had felt that different situations had been stressful the past month (40). The PSS is measured on a five-points likert scale ranging from zero (never) to four (very often). A high score on the questionnaire indicates experiences of high level of psychological stress. Scores of 20 or higher are considered high stress. The maximum possible score on the PSS is 40 (40, 51).

#### Blood Pressure

BP was measured using an OBH blood pressure monitor (model 4600), which uses an oscillometric measure method. The device measures the systolic and diastolic blood pressure and the pulse. Participants' blood pressure was measured at three different times, when they had been relaxed for at least 10 min. The mean blood pressure of these three times was used [e.g., (52–54)].

#### Generalized Anxiety Disorder Scale—Revised

GADS-R measures the role of metacognitions in relation to generalized anxiety. Two of the factors measured by the questionnaire are particularly relevant to this study: (1) *Positive metacognitive beliefs*, which indicates to what degree the respondent believes that deliberate thinking is useful; (2) *Negative metacognitive beliefs*, which indicates the degree to which the respondent believes that deliberate thinking/worry is uncontrollable and physically dangerous. Additionally, the degree of avoidance and use of coping strategies was measured using the same scale (27). Of the GADS-R, weekly time spent worrying is measured on scale from 0 (“no time”) to 8 (“all the time”); how often 9 coping behaviors and 6 avoidance behaviors are enacted in a week are measured on the same scale from 0 to 8 and summed; negative and positive metacognitive beliefs related to worry are measured on a scale from 0 (“I do not believe this at all”) to 100 (“I'm completely convinced this is true”).

#### Becks Depression Inventory II

BDI-II *was* used to measure depressive symptoms in youth and adults. BDI-II is a self-report questionnaire consisting of 21 items. The scores are categorized in four categories ranging from mild to severe depression (41).

#### Structured Clinical Interview for DSM-IV Axis-1 Disorder

SCID-1 was used as an assessment and screening for the major DSM-IV axis-1 disorders before the start of the study (42, 43).

### Data Analysis

#### Data Analysis Plan

Data analysis has been conducted in two steps: (1) a series of repeated measures analysis of variance (ANOVA) and subsequent protected paired-samples *t*-tests analyzed the general effect of treatment on the whole sample (testing for H1, H2, H3, and H4); and (2) a clinically significant change analysis tested for clinical and statistical improvement of general mental health and reduction of perceived stress of each client (testing for H5). Afterwards, secondary outcome variables were assessed by a series of repeated-measures ANOVAs to test for the general effects of the treatment on such variables.

#### Data Analysis for Main Results

To test for our main research hypotheses (effect of the treatment on the four primary outcome variables), we conducted a series of repeated-measures ANOVAs. Given the small sample size (*N* = 4), we approached this analysis with a 3-fold rationale: (1) we tested for significant differences in GHQ-30, PSS, Systolic, and Diastolic Blood Pressure (respectively, SBP and DBP) at baseline (average of measures across the baseline weeks measures), end of treatment (last week of treatment of the client) and follow-up (average of measures), in order to avoid any missing data; (2) to reduce the risk of Type I error we followed the approach suggested by Oberfeld and Franke (55), and conducted a series of more conservative paired *t*-test (56) to specifically test for significant differences between baseline and end of treatment, as well as non-significant differences between end of treatment and follow up; (3) we calculated the effect size following Cohen's *d* formula for equal sample sizes (56, 57):

$$\begin{array}{l} d=\;\frac{\mu_{1}-\;\mu_{2}}{\sigma } \end{array}$$

## Results

According to the data analysis plan, results are presented in three sections: main results, clinical significant change and secondary outcome variables.

### Main Results

#### General Mental Health

The first analysis concerns the effect of treatment on participants' general health (GHQ-30). Results show a significant effect of treatment on clients' general health, *F*_(2, 6)_ = 15.08, *p* = 0.005, $\eta_{p}^{2}$= 0.97. Two subsequent protected *t*-tests have been conducted. Confirming hypotheses H1a and H1b, GHQ-30 score significantly dropped (indicating increase in clients' general health) from baseline (*M* = 13.20; *SD* = 4.59) to end of treatment [*M* = 1.75; *SD* = 3.5; *t*_(6)_ = 4.68; *p* = 0.003; *d* = 3.82], and then remained stable in the follow up [*M* = 1.37; *SD* = 1.60; *t*_(6)_ = 0.15; *p* = 0.88; *d* = 0.13]. General health of participants improved throughout the therapy and then remained stable after it.

#### Perceived Stress

The second analysis concerns participants' perceived stress (PSS). Results show a significant effect of treatment on clients' time spent worrying, *F*_(2, 6)_ = 34.52, *p* = 0.001, $\eta_{p}^{2}$= 0.92. Two subsequent protected *t*-tests have been conducted. Confirming hypotheses H2a and H2b, PSS score significantly dropped (indicating decrease in perceived stress) from baseline (*M* = 25.25; *SD* = 2.87) to end of treatment [*M* = 7.00; *SD* = 3.46; *t*_(6)_ = 7.04; *p* < 0.001; *d* = 5.75], then remained stable in the follow up [*M* = 6.25; *SD* = 3.12; *t*_(6)_ = 0.29; *p* > 0.78; *d* = 0.23]. Clients' perceived stress decreased throughout the therapy and then remained stable and after it.

#### Systolic Blood Pressure and Diastolic Blood Pressure

The third analysis concerns participants' systolic (SBP) and diastolic (DBP) blood pressure, measured by OBH blood pressure monitor. Results show a significant effect of treatment on clients' SBP, *F*_(2, 6)_ = 10.31, *p* = 0.011, $\eta_{p}^{2}$= 0.77. Two subsequent protected *t*-tests confirm hypotheses H3a and H3b: SBP significantly dropped (indicating decrease in physiological stress) from baseline (*M* = 139.5; *SD* = 18.65) to end of treatment [*M* = 129.00; *SD* = 18.92; *t*_(6)_ = 2.47; *p* = 0.048; *d* = 2.02], then remained stable in the follow up [*M* = 120.25; *SD* = 14.50; *t*_(6)_ = 2.06; *p* > 0.085; *d* = 1.68].

Furthermore, results show a significant effect of treatment on clients' DBP, *F*_(2, 6)_ = 16.12, *p* = 0.004, $\eta_{p}^{2}$= 0.84. Two subsequent protected *t*-tests confirm hypotheses H4a and H4b: DBP significantly dropped (indicating decrease in physiological stress) from baseline (*M* = 88.5; *SD* = 9.57) to end of treatment [*M* = 79.25; *SD* = 11.70; *t*_(6)_ = 3.63; *p* = 0.011; *d* = 2.97], then remained stable in the follow up [*M* = 74.25; *SD* = 8.96; *t*_(6)_ = 1.96; *p* > 0.097; *d* = 1.60]. Clients' SBP and DBP decreased throughout the therapy and then remained stable and after it.

#### Return to Work and Qualitative Analysis of Feasibility and Acceptability of Treatment

Although not originally hypothesized, 100% of clients returned to work after treatment, and they continued working during follow up.

Furthermore, our clients showed great acceptability of the treatment, as demonstrated by their comments here reported:

“*I have learned to leave my thoughts alone and it makes me far less stressed”*—RL

“*I worry a lot less, feel relieved, calm and happy. It is unbelievable that I have learnt so much in such short time –something I have spent my whole life learning”*—AJ.

“*I didn't imagine that the therapy would have such a big impact. My focus is now not to give my negative thoughts energy, and that doing-nothing has made me very happy. (…) It is chaotic at work, I am alone in my team, all my colleagues are off work sick with stress, but I don't worry about it and I don't feel stressed”*—SH.

“*I went from a bedbound life to an active life through this progress. I thought before that my body was decomposed because of too hard work. This was a faulty belief. Stress doesn't make me sick. Thoughts and worries don't make me sick. I become sick if I think that thoughts and worries are me. That I am my thoughts and that they are the truth. Now I just let my thoughts pass. Being highly stressed is something I can select and deselect”*—TM.

Therefore, from return-to-work rates and the high acceptability of the treatment, it seems that our proposed solution can be effective and feasible.

### Clinically Significant Change

We conducted clinical significant change analysis (58) for each client. The cut-off score (*cut-off*_*r*_) can be calculated by defining a population-specific interval for dysfunctionality, which is two standard deviations from the dysfunctional population's average (58). If the client's score at end of treatment moves out of this interval toward functionality, then a clinical significant change has occurred (58). The whole set of individual scores of each client is reported in Table 1A–D; also, we report the average scores in Table 2.

**Table 1:** Individual scores of each client on primary and secondary outcome variables.

**Individual scores**	**Week**	**BDI-II**	**GHQ-30**	**Time spent worrying**	**Maladaptive coping strategies**	**Avoidance behaviors**	**Negative beliefs**	**Positive beliefs**
**(A) Client: SH**								
Baseline	−6	–	–	–	–	–	–	–
	−5	–	–	–	–	–	–	–
	−4	–	–	–	–	–	–	–
	−3	14	7	2	2	0	20	12.5
	−2	12	7	0	0	0	50	12.5
	−1	16	9	4	0	5	50	0
Treatment	1	19	14	2	0	0	250	100
	2	17	17	6	12	0	350	100
	3	20	16	4	5	0	200	150
	4	11	8	8	18	0	350	200
	5	15	3	1	7	0	150	150
	6	19	14	5	6	0	50	0
	7	12	7	2	8	0	0	0
	8	7	0	1	2	0	110	0
	9	8	0	1	2	0	0	0
	10	–	–	–	–	–	–	–
Follow-up	3 Months	14	3	4	10	0	100	0
	6 Months	8	2	1	0	0	50	0
**(B) Client: RL**								
Baseline	−6	–	–	–	–	–	–	–
	−5	–	–	–	–	–	–	–
	−4	22	19	8	33	4	78	32.5
	−3	16	19	7	25	4	78	32.5
	−2	15	19	7	25	4	78	32.5
	−1	21	18	8	33	4	78	32.5
Treatment	1	18	18	8	27	4	440	200
	2	13	17	8	27	4	400	150
	3	18	17	8	32	2	320	100
	4	9	15	3	17	0	210	80
	5	6	5	4	7	0	170	140
	6	3	1	1	4	0	55	0
	7	3	0	1	0	0	30	50
	8	2	0	0	0	0	0	30
	9	6	5	0	6	0	0	5
	10	–	–	–	–	–	–	–
Follow-up	3 Months	4	5	2	6	0	30	10
	6 Months	1	1	1	2	0	0	20
**(C) Client: TM**								
Baseline	−6	–	–	–	–	–	–	–
	−5	16	13	6	33	23	84	20
	−4	13	12	3	40	17	78	20
	−3	11	11	3	31	19	76	27.5
	−2	15	15	3	31	16	86	27.5
	−1	15	10	3	30	17	70	27.5
Treatment	1	10	6	3	27	20	400	180
	2	10	10	4	35	17	420	180
	3	9	7	3	39	17	460	120
	4	8	4	2	18	15	430	110
	5	3	0	1	8	10	220	140
	6	0	1	2	11	8	135	50
	7	0	0	1	11	5	30	10
	8	0	0	1	0	0	0	0
	9	–	–	–	–	–	–	–
	10	–	–	–	–	–	–	–
Follow-up	3 Months	1	0	0	0	0	0	0
	6 Months	2	0	3	17	7	230	30
**(D) Client: AJ**								
Baseline	−6	20	13	6	18	7	64	25
	−5	22	17	7	12	8	68	12.5
	−4	18	13	3	15	2	66	25
	−3	21	13	3	12	0	56	37.5
	−2	22	12	4	17	4	72	27.5
	−1	21	17	6	12	8	72	25
Treatment	1	13	16	1	18	0	340	170
	2	22	19	4	28	28	380	150
	3	23	21	4	25	7	380	60
	4	21	11	2	28	3	370	140
	5	9	11	2	5	0	50	0
	6	14	8	4	9	2	0	20
	7	10	9	2	2	0	30	20
	8	10	7	1	0	0	10	0
	9	4	0	1	0	0	0	0
	10	3	1	1	0	0	0	0
Follow-up	3 Months	6	0	0	0	0	0	0
	6 Months	–	–	–	–	–	–	–

**Table 2:** Average scores of all clients on primary and secondary outcome variables.

**Average scores**		***N*** **=** **4**						
	**Week**	**BDI-II**	**GHQ-30**	**Time spent worrying**	**Maladaptive coping strategies**	**Avoidance behaviors**	**Negative beliefs**	**Positive beliefs**
Baseline	−6	**–**	**–**	**–**	**–**	**–**	**–**	**–**
	−5	**–**	**–**	**–**	**–**	**–**	**–**	**–**
	−4	**–**	**–**	**–**	**–**	**–**	**–**	**–**
	−3	15.5	12.5	3.8	17.5	5.8	57.5	27.5
	−2	16.0	13.3	3.5	18.3	6.0	71.5	25.0
	−1	18.3	13.5	5.3	18.8	8.5	67.5	21.3
Treatment	1	15.0	13.5	3.5	18.0	6.0	357.5	162.5
	2	15.5	15.8	5.5	25.5	12.3	387.5	145.0
	3	17.5	15.3	4.8	25.3	6.5	340.0	107.5
	4	12.3	9.5	3.8	20.3	4.5	340.0	132.5
	5	8.3	4.8	2.0	6.8	2.5	147.5	107.5
	6	9.0	6.0	3.0	7.5	2.5	60.0	17.5
	7	6.3	4.0	1.5	5.3	1.3	22.5	20.0
	8	4.8	1.8	0.8	0.5	0.0	30.0	7.5
	9	–	–	–	–	–	–	–
	10	–	–	–	–	–	–	–
Follow-up	3 Months	6.3	2.0	1.5	4.0	0.0	32.5	2.5
	6 Months	–	–	–	–	–	–	–

*Average scores are computed for complete data only*.

#### General Mental Health

First, a *general* cut-off score of 5 has been identified in the literature for the GHQ-30 (47): after treatment and at follow-up, 4 out of 4 of clients' scores were below Goldberg and Williams' cut-off score of 5 for GHQ-30, clinically significant change in general mental health. However, for a more conservative analysis, a *specific* clinical significant change was also tested in each client by calculating the population-specific cut-off score (58). The specific GHQ-30 cut-off score is therefore computed as it follows: GHQ-30 baseline: *M* = 13.20, *SD* = 4.60, *cut-off*_*r*_ = 4.00 (*M*-2^*^SD)—similar to Goldberg and Williams' (47) cut-off score. Results show that at end of therapy: (a) all clients reported a clinically significant change compared to the *general* cut-off; (b) 3 out of 4 clients reported a clinically significant change compared to the population *specific* cut-off; and (c) all clients maintained (or improved) the clinical significant change at follow-up. Therefore, by the end of treatment and, critically, at follow-up too, the clients no longer report a dysfunctional mental health.

#### Perceived Stress

The PSS is not a diagnostic measure, hence a general cut-off score of clinical significance does not exist (40, 51, 59). Yet, previous research (51) has identified normative average PSS score for white adults (*M* = 15.70). Again, for a more conservative analysis, the PSS population *specific* cut-off score is also computed: PSS baseline: *M* = 25.25, *SD* = 2.87, *cut-off*_*r*_ = 19.51 (M-2^*^SD). Results show that at end of therapy: (a) all clients reported a significant change compared to the normative average; (b) all clients reported a significant change compared to the *specific* cut-off; and (c) all clients maintained (or improved) the significant change at follow-up. The clients, by the end of treatment and, critically, at follow-up too, significantly reduced their perceived stress at below-average levels.

Therefore, the clinically significant change analysis shows that a significant change has been reached in each client, confirming H5.

### Secondary Outcome Variables

We conducted a series of repeated-measures ANOVAs to test for the general effects of the treatment on the secondary outcome variables. According to previous research [e.g., (27, 32, 33)], it is worth to explore how the following symptoms—secondary outcome variables here—have responded to the present treatment: generalized anxiety disorder symptoms (operationalized by the GADS-R scores of time spent worrying, positive and negative metacognitive beliefs, maladaptive coping strategies and avoidant behaviors) and depressive symptoms (operationalized by the BDI-II). The whole set of results are presented in Table 3. Overall, confirming H5: clients' anxiety and depressive symptoms significantly decreased throughout the therapy and then remained stable and after it. Specifically, this effect holds true for all the GADS-R sub-scales (time spent worrying, positive beliefs, maladaptive coping strategies and avoidance behaviors) except one (negative beliefs), and for the BDI-II scale (depressive symptoms).

**Table 3:** Repeated measures ANOVA and protected t-tests testing change in secondary outcome variables (anxiety symptoms and metacognitions in the GADS-R, and depressive symptoms in the BDI-II).

**Measure**		**Baseline (T1)**	**End of treatment (T2)**	**Follow-up (T3)**	**Onmibus effect**		**T1–T2**		**T2–T3**	
		**M(SD)**			***F (df)***	**Part. Eta sq**.	***t (df)***	***d***	***t (df)***	***d***
**GADS-R**
	Time spent worrying	4.48 (2.32)	0.75 (0.50)	1.37 (1.03)	5.48 (2, 6)	0.65	3.09^*^ (6)	−2.52	0.51 (6)	0.42
	Negative beliefs	65.78 (18.11)	30.0 (53.54)	51.25 (53.44)	0.61 (2, 6)	0.17	–		–	
	Positive beliefs	22.69 (10.21)	7.50 (15.00)	7.50 (8.66)	5.33 (2, 6)	0.64	2.82^*^ (6)	−2.31	–	
	Maladaptive coping strategies	19.25 (14.76)	0.50 (1.00)	4.37 (3.50)	5.29 (2, 6)	0.64	3.08^*^ (6)	−2.51	0.64 (6)	0.52
	Avoidance behaviors	7.22 (7.57)	0 (0)	0.87 (1.75)	3.94 (2, 6)	0.57	2.57^*^ (6)	−2.1	0.31 (6)	0.25
**BDI-II**
	Depressive symptoms	17.00 (3.56)	4.25 (3.50)	6.25 (5.56)	12.75 (2, 6)	0.81	4.69^**^ (6)	−3.83	0.74 (6)	0.6

* *p < 0.05;*

** *p < 0.01*.

*Dashes indicate that the analysis was not performed given that the omnibus effect was not significant or the t-test was trivial given that means to be compared were identical*.

Summarizing, results confirm the prediction that treatment, fostering change in metacognitive knowledge and strategies, can reduce work-related stress. Results show an overall improvement of clients' general health and, critically, the reduction of clients' self-reported *and* physiological stress (both systolic and diastolic blood pressure). In addition, results show improvements in secondary outcome variables, such as reduction in anxiety and depressive symptoms.

## General Discussion

In the present study we examined the effects of MCT in the treatment of work-related stress. Four clients have been included in the study, undergoing a period of baseline screening of primary and secondary outcome psychological variables (general mental health, perceived stress, blood pressure, generalized anxiety disorder and depression), the treatment itself, and follow-up measurements of such variables. Confirming H1-2, results show significant improvement in clients' general mental health and stress at end of treatment, and, critically, these effects remained stable at follow-up. Furthermore, confirming H3-4, effects are confirmed for the physiological measures of stress, namely systolic and diastolic blood pressure. Importantly, results are confirmed in terms of individual significant clinical change in each client, confirming H5. Furthermore, these effects are confirmed for secondary outcome variables too, namely anxiety and depressive symptoms.

Taken together, these results corroborate the idea that MCT can effectively be used to reduce work-related stress. MCT may affect the clients' stress cognitive style, including worries and metacognitive beliefs, which are involved in the development and the maintenance of emotional disorders (29). Critically, in our study, general mental health, perceived stress and blood pressure decreased significantly during treatment and remained stable at follow up, suggesting an overall reduction of clients' psychological and physiological stress (51, 54). Also, amount of worries, positive metacognitive beliefs, maladaptive coping strategies and avoidance behaviors decreased significantly during treatment and follow-up, while depressive symptoms decreased as well, respectively, suggesting an overall reduction of the cognitive attentional syndrome and of stress' comorbid symptoms (27, 31–33). These improvements happened over a short treatment period of eight-to-ten sessions and remained stable over a follow-up period of 3–6 months. Furthermore, during the treatment, while no clients reported worsening in symptoms or stress, our qualitative examination of client satisfaction also showed that the clients regarded metacognitive therapy as an acceptable, effective, and feasible treatment for work-related stress.

### Transdiagnostic Effectiveness of Metacognitive Therapy

Clients with impairing stress often experience comorbidity such as anxiety or depression (27, 31, 32), which can be presumed to impede and prolonged treatment (60). Although the therapy focused on stress and not on the possible secondary outcome variables, all four clients showed a lower result on the anxiety and depression scales (GADS-R and BDI-II) at the end of therapy. This indirect result supports the hypothesis of MCT as a transdiagnostic model of treatment (27, 28, 30, 31, 37, 61). Thus, if MCT should be used as a transdiagnostic model of treatment in the future, it is recommended to make a targeted effort to work with the specific maintenance processes in the comorbid disorder (27, 28), which was not the case in this study that only focused on work-related stress symptoms.

### Limitations and Future Developments

Some limitations should be considered when interpreting these results. This study is based on data from four clients, limiting the possibility to generalize the treatment effects and to discuss possible effects of extraneous variables (such as gender, type of work, pandemic situation): however, (a) parametric statistical tests (repeated-measures ANOVAs) allowed us to test for the omnibus effect of treatment on different variables and then, to reduce the risk of Type I error (55), we tested our main hypotheses through a series of more conservative analyses [protected paired *t*-test; (56)]; (b) we conducted a clinical significant change analysis (58), which showed that all clients' improved on individually on the primary outcome variables; (c) gender was balanced in our sample (50/50) and age span from 26 to 56 y, showing that our intervention was effective for young to middle-aged men and women alike.

Other strategies commonly used in MCT could exert a confounding effect [similarly to limitations in other case studies; e.g., (35)] as in all therapies it is not possible to isolate the effect of treatment strategies on the results: however, (a) our study method minimizes effects of spontaneous improvement, client expectations and repeated measures effect on the results by the use of a multiple baseline assessment; and (b) parametric statistic approach screens out non-specific factors, such as a better or worse therapeutic alliance.

The use of self-report measures for non-clinical factors such as perceived stress might induce social desirability which can affect the results: however, although the use of self-report measures such as the PSS used here is widely used in clinical psychology research (51), the present study also included an objective measurement of the clients' physiological stress (systolic and diastolic blood pressures), which is not affected by the clients' subjective attitudes, nor by potential social desirability. The consistency of psychological and physiological measures of stress, the change of such measures after treatment, as well as their stability at follow up, show a clear pattern of results.

## Conclusion

The results of this study suggest that dysfunctional metacognitive mechanisms could be involved in the maintenance of work-related stress and that MCT could a suitable, cost-effective treatment for work-related stress. MCT is here associated with improvements in work-related stress symptoms (self-reported and blood pressure) and in general mental health, just after 8–10 sessions, which persisted at follow-up. This took place at the same time as maintaining strategies were changed, which is the aim of MCT (27). Consistent with previous research (31), here we show that MCT principles help reducing stress and mitigating its maintaining factors. To our knowledge, the present study is one of the first promising application of MCT on work-related stress. However, a specific protocol for such application has not been developed yet. Based on these considerations, and given the growing concerns of governments on mental health issues (62) and the mounting amount of illness associated to work-related stress (63) further worsened by the COVID-19 pandemic (13, 14, 19), a specific metacognitive model for stress needs to be developed, and larger, randomized controlled studies need to be conducted to further develop the efficacy of MCT on work-related stress. Various stakeholders should consider the promising results of our study, and eventually benefit from the feasibility, time and cost effectiveness and sustainability of MCT-based interventions to reduce work-related stress.

## Data Availability Statement

The raw data supporting the conclusions of this article will be made available by the authors, without undue reservation.

## Ethics Statement

Ethical review and approval was not required for the study on human participants in accordance with the local legislation and institutional requirements. The patients/participants provided their written informed consent to participate in this study. Written informed consent was obtained from the individual(s) for the publication of any potentially identifiable images or data included in this article.

## Author Contributions

SD: writing—original draft preparation, writing—review and editing, formal analysis, and visualization. MT: writing—original draft preparation, conceptualization, methodology, investigation, and data curation. PC: supervision. All authors contributed to the article and approved the submitted version.

## Conflict of Interest

The authors declare that the research was conducted in the absence of any commercial or financial relationships that could be construed as a potential conflict of interest.
